# Status-Dependent Vasotocin Modulation of Dominance and Subordination in the Weakly Electric Fish *Gymnotus omarorum*

**DOI:** 10.3389/fnbeh.2018.00001

**Published:** 2018-01-18

**Authors:** Rossana Perrone, Ana C. Silva

**Affiliations:** ^1^Unidad Bases Neurales de la Conducta, Instituto de Investigaciones Biológicas Clemente Estable, Montevideo, Uruguay; ^2^Laboratorio de Neurociencias, Facultad de Ciencias, Universidad de la República, Montevideo, Uruguay

**Keywords:** agonistic behavior, vasotocin modulation, social status, electric signals, electric fish

## Abstract

Dominant-subordinate status emerges from agonistic encounters. The weakly electric fish, *Gymnotus omarorum*, displays a clear-cut example of non-breeding territorial aggression. The asymmetry in the behavior of dominants and subordinates is outstanding. Dominants are highly aggressive and subordinates signal submission in a precise sequence of locomotor and electric traits: retreating, decreasing their electric organ discharge rate, and emitting transient electric signals. The hypothalamic neuropeptide arginine-vasotocin (AVT) and its mammalian homolog arginine-vasopressin, are key modulators of social behavior, known to adapt their actions to different contexts. By analyzing the effects of pharmacological manipulations of the AVT system in both dominants and subordinates, we show evidence of distinct status-dependent actions of AVT. We demonstrate an endogenous effect of AVT on dominants' aggression levels: blocking the V1a AVT receptor induced a significant decrease in dominants' attack rate. AVT administered to subordinates enhanced the expression of the electric signals of submission, without affecting subordinates' locomotor displays. This study contributes a clear example of status-dependent AVT modulation of agonistic behavior in teleosts, and reveals distinctive activation patterns of the AVT system between dominants and subordinates.

## Introduction

Agonistic behavior, the social behavior related to conflict situations between conspecifics, has shaped sociality across evolution (Lorenz, 1963; King, 1973). Conflicts arise because animals compete for different resources (territory, food, mates, breeding sites, etc.) and they are solved when one individual keeps the resource (dominant) and the other loses it (subordinate). Though the behavioral traits displayed during contests may be extremely diverse across species, agonistic encounters always follow three phases: evaluation, contest, and post-resolution, with overt aggression usually occurring during the contest phase (Nelson, 2006; Summers and Winberg, 2006). This stable profile is the result of a complex evaluation process among contenders that allows them to make the decision of escalating or giving up the contest (Maynard Smith and Parker, 1976). As a result, a clear status-dependent asymmetry in the behavior of contenders is observed during the post-resolution phase, which necessarily relies on distinctive neuroendocrine mechanisms that control the emergence of either dominance or subordination.

The hypothalamic neuropeptide arginine-vasotocin (AVT) and its mammalian homolog, arginine-vasopressin (AVP) are key modulators of social behavior (Goodson and Bass, 2001; Albers, 2015). AVT/AVP modulation of social behavior varies between species, sexes, physiological states, phenotypes, and social contexts (Insel and Young, 2000; Goodson et al., 2009; Godwin and Thompson, 2012; Caldwell, 2017; Johnson and Young, 2017). These neuropeptide systems have also been associated with social status; for example, dominance is related with a distinctive distribution of AVP receptors within the social brain (Cooper et al., 2005; Filby et al., 2010; Lema et al., 2015). Furthermore, a differential pattern of activation of AVT/AVP neurons between dominants and subordinates has been reported in different vertebrates (Ferris et al., 1989; Larson et al., 2006; Greenwood et al., 2008; Hattori and Wilczynski, 2009; Godwin and Thompson, 2012; Qiao et al., 2014; Lema et al., 2015; Teles et al., 2016; Terranova et al., 2016; Pouso et al., 2017). Pharmacological manipulations have also contributed indirect evidence for status-dependent actions of these neuropeptides (Goodson and Bass, 2000; Backström and Winberg, 2009), although there are few studies that explore these actions by comparing the same treatments on both dominants and subordinates (Semsar et al., 2001; Goodson et al., 2009; Huffman et al., 2015).

Weakly electric fish are valuable model systems for the study of agonistic behavior and its neuromodulation given that they display conspicuous social electric signals in addition to locomotor traits, which are generated by a very well-known electromotor circuit (Stoddard, 2002; Caputi et al., 2005). The electric organ discharge (EOD) carries information about an individual's species identity, sex, and physiological state, coded both in its rate and waveform (Caputi et al., 2005). Many studies have reported distinctive agonistic electric displays (either produced by dominants or subordinates) in several species of South American freshwater electric fish (Westby G., 1975; Westby G. W. M., 1975; Hagedorn and Zelick, 1989; Hupé and Lewis, 2008; Triefenbach and Zakon, 2008; Perrone et al., 2009; Fugère et al., 2010). In particular, EOD rate has been reported as indicator of dominance (Hopkins, 1974; Westby G., 1975; Hagedorn and Carr, 1985; Zakon et al., 1991; Triefenbach and Zakon, 2008; Fugère et al., 2010); the cessation in the emission of electric signals (offs) has been interpreted as a submissive signal; (Hopkins, 1974; Westby G., 1975; Hagedorn and Carr, 1985; Zakon et al., 1991; Triefenbach and Zakon, 2008), and chirps (brief, transient EOD modulations) can either signal threat or submission depending on the species (Black-Cleworth, 1970; Westby G. W. M., 1975; Hagedorn and Zelick, 1989; Hupé et al., 2008; Triefenbach and Zakon, 2008; Perrone et al., 2009; Batista et al., 2012; Perrone and Silva, 2016).

*Gymnotus omarorum* (Richer-de-Forges et al., 2009) displays a clear-cut example of pure territorial aggression (Batista et al., 2012; Silva et al., 2013; Jalabert et al., 2015; Zubizarreta et al., 2015; Quintana et al., 2016). During the non-breeding season, when gonads are regressed, and no reproductive motivation is expected to drive competition, males and females of this sexually monomorphic species fiercely defend territories in intrasexual and intersexual dyadic encounters. The asymmetry in the behavior of dominants and subordinates of *G. omarorum* is outstanding. While dominants are highly aggressive even after the conflict is clearly solved, subordinates signal submission in a precise sequence of locomotor and electric traits (Batista et al., 2012; Quintana et al., 2016). This model system has two main advantages to explore the neuroendocrine mechanisms involved in the emergence of the dominant-subordinate status. First, it is independent of gonadal steroid hormones (Jalabert et al., 2015), which provides a clean hormonal scenario to evaluate the action of other candidate modulators. Second, it has a rich repertoire of easily accessible and well-understood locomotor and electric displays (Batista et al., 2012; Quintana et al., 2016), which provides multiple indicators and putative targets to explore the action of different modulators.

In this study, we focus on the role of AVT on the agonistic behavior of *G. omarorum* by analyzing the effects of pharmacological manipulations of the AVT system in both dominants and subordinates. Our results contribute to the understanding of the complexity of the role of hypothalamic neuropeptides in the control of social behavior as we show evidence of distinct status-dependent actions of AVT. While AVT modulates the intensity of aggression and the readiness to attack in dominants, it enhances the electric signaling of submission in subordinates.

## Materials and methods

We used non-breeding adult *G. omarorum* (Richer-de-Forges et al., 2009), that ranged from 15.5–31.5 cm in body length and 9–91 g in body weight. Sex in *G. omarorum* is not externally apparent (neither morphologically nor electrophysiologically) and was determined after the behavioral experiments by gonadal inspection; the sex ratio was around 1:1 (45 males and 41 females). All experiments were performed during the non-breeding season (May–July).

Fish were collected as described elsewhere (Silva et al., 2003). *Gymnotus omarorum* were collected in the freshwater lagoon Laguna del Sauce (34°51′S, 55°07′W, Department of Maldonado, Uruguay), and housed in individual mesh compartments in 500-l outdoor tanks. The fish were housed in outdoor tanks for at least 10 days before the behavioral experiments. All environmental variables were kept within the normal range exhibited in the natural habitat in the non-breeding season. Water temperature ranged from 8 to 21°C, and natural photoperiod ranged from LD10:14 to LD11:13. Water conductivity was adjusted and always maintained below 200 μS/cm by the addition of deionized water. Aquatic plants (*Eichhornia crassipes, Pistia stratiotes, Salvinia* sp.) covered the surface of the water and provided shelter for the fish. Fish were fed with *Tubifex tubifex* once a week.

Electric fish collection for experimental purposes was authorized by DINARA (National Direction of Aquatic Resources) and MGAP (Ministry of Agriculture and Fisheries), resolution No. 065/2004. All experimental procedures complied with ASAP/ABS Guidelines for the Use of Animals in Research and were approved by our institutional ethical committee (Comisión Bioética, Instituto Clemente Estable, MEC, 007/02/2010).

### Behavioral recording station

Fish were placed in an experimental setup that allowed simultaneous video and electric recordings as described elsewhere (Silva et al., 2007). The experimental tanks, four 50-l glass aquaria (55 × 40 × 25 cm) were fitted with two pairs of orthogonal electrodes attached to each tank wall. The day–night cycle and the physicochemical parameters (water temperature, conductivity, and pH) of indoor tanks matched those of the outdoor housing tanks. All the experiments were performed in total darkness illuminated by an array of infrared LEDs (L-53F3BT, Fablet&Bertoni Electronics) located above the tank. An infrared-sensitive video camera (SONY CCD-Iris and RoHS CCD Digital Video Camera) was focused on the bottom of the tank. Electric signals of freely moving fish were detected by two pairs of fixed electrodes, connected to two high-input impedance amplifiers (FLA-01, Cygnus Technologies Inc.). Images and electric signals were captured by a video card (Pinnacle Systems, PCTV HD pro stick) and stored in the computer for further analysis. The fish remained in the recording tank at constant temperature (16–20°C) for 4–5 h before the experiments in separate compartments in which each contender could perceive a distorted and low-amplitude signal from the other fish.

### Behavioral experimental procedures

Although territorial agonistic behavior occurring all year round in this species, all behavioral experiments were performed during the non-breeding season (occurs during the Austral fall-winter time) to avoid any other type of agonistic interactions related to reproduction. We tested the territorial aggression of *G. omarorum* in experimental conditions in which territory is the only resource that individuals fight for, providing symmetric resources and resource values for both contestants: equally-sized plain territory, same residence time, and the same previous experience (Batista et al., 2012). As weight difference is a proxy of dominance (Batista et al., 2012), we used dyads in which body weight difference ranged from 5 to 20% (*n* = 43) to predict the contest outcome. In all experiments, a removable glass gate was raised 5 min after sunset, and fish were separated 10 min following conflict resolution. As the non-breeding territorial aggression of *G. omarorum* is sex-independent (Batista et al., 2012), we used both inter-sexual and intrasexual dyads.

### Pharmacological administration

The 8- arginine-vasotocin (AVT) and its competitive antagonist, the Manning Compound (MC: [Pmp1,Tyr(OMe)2,Arg8] Vasopressin) were purchased from American Peptide Company. We evaluated the effects of AVT (1 μg/g body weight of a 1 μg/μl saline solution) and MC (2 μg/g of a 1 μg/μl saline solution) in potential dominants and subordinates of *G. omarorum* by intraperitoneal administration prior to the agonistic encounter. In all experiments, the other animal of the dyad was also intraperitoneally injected with the same volume of a physiological saline solution. Both pharmacological treatments were administered 30 min before the agonistic encounter, based on previous findings that show the maximum effect of AVT and MC at such doses occurs 30 min after the injection (Perrone et al., 2010). In the few cases in which the expected outcomes were reversed (Table 1), we reassigned the animals to the corresponding experimental group; i.e., the subordinate animal was included in the subordinate group even in the cases in which it was the largest animal of the dyad. We used 5 experimental groups: (a) control dyads (*n* = 11); (b) dyads in which the dominants were treated with AVT (*n* = 11); (c) dyads in which the subordinates were treated with AVT (*n* = 9); (d) dyads in which the dominants were treated with MC (*n* = 6); and (e) dyads in which the subordinates were treated with MC (*n* = 6).

**Table 1:** Locomotor parameters of agonistic encounters under different treatments.

	**Control *n* = 11**	**AVT *n* = 11**	**MC *n* = 6**	**Overall comparison** ***p*****-values test**	
**DOMINANTS**					
*Outcome* (% big fish won)	90.91	70	83.33	>0.05	(χ^2^)
*Contest duration* (s)	222.8 (±71.8)	227 (±165)	134 (±55.5)	>0.05	(K-W)
*First attack latency* (s)	22 (±11)	27.50 (±13.5)	59.15 (±22)	>0.05	(K-W)
*Contest attack rate* (n/s)	0.12 (±0.04)	0.08 (±0.05)	**0.06 (**±0.01)^*^	**0.05**	(K-W)
*Post-resolution attack rate* (n/s)	0.04 (±0.02)	0.05 (±0.02)	0.05 (±0.03)	>0.05	(K-W)
	**Control *n* = 11**	**AVT *n* = 9**	**MC *n* = 6**	**Overall comparison ***p***-values test**	
**SUBORDINATES**					
*Outcome* (% big fish won)	90.91	54.54	85.71	>0.05	(χ^2^)
*Contest duration* (s)	222.8 (±71,8)	150 (±47.5)	221 (±157.5)	>0.05	(K-W)
*first Attack latency* (s)	31 (±15.3)	24 (±13)	20 (±8.6)	>0.05	(K-W)
*Contest attack rate* (n/s)	0.04 (±0.02)	0.05 (±0.02)	0.05 (±0.03)	>0.05	(K-W)
*Post-resolution attack rate* (n/s)	0.0 (±0.0)	0.0 (±0.0)	0.0 (±0.0)	>0.05	(K-W)

*Overall comparisons were done by Chi-square test 3 × 2 (χ^2^, outcome) and Kruskal–Wallis test followed by the post-hoc Dunn test (K-W, other parameters)*.

* *p ≤ 0.05. Dominants' attack rate: Kruskal–Wallis test, p = 0.05, post-hoc Dunn test, Control vs. AVT, p = 0.29; Control vs. MC, p = 0.07; AVT vs. MC, p > 0.99*.

### Behavioral data processing

#### Locomotor displays

We analyzed the locomotor displays of the tested individuals to identify the three phases of the agonistic encounter following Batista et al. (2012): (a) evaluation phase (pre-contest): from time 0 (gate removal) to the occurrence of the first attack; (b) contest phase: from the first attack to conflict resolution (resolution time); and (c) post-resolution phase (post-contest): 600 s after conflict resolution. Contest resolution was established when we observed the third consecutive retreat of one fish without attacking back. This criterion unambiguously defined subordination status; subordinates were never observed to change their status in the following 600 s of interaction.

We measured the following locomotor parameters in all the experiments: latency to the first attack (including nips, nudges, bites), contest duration, contest attack rate (number of attacks/contest duration in seconds) of dominants and subordinates; and post-resolution attack rate (number of attacks/600 s) of dominants and subordinates.

#### Electric signals

EOD rate was calculated as the mean instantaneous frequency in 5–10 s samples obtained from the evaluation and post-resolution phases. The EOD rate change index was calculated as [(EOD rate in the post-resolution phase)-(EOD rate in the evaluation phase)]/(EOD rate in the evaluation phase) in percentage. Positive values of the index mean an increase in the EOD rate, and negative values of the index mean a decrease in the EOD rate in the post-resolution phase. This index was calculated for all control (*n* = 11) and pharmacologically modulated dyads (*n* = 32).

We measured the occurrence and timing of offs (interruptions of EOD emission), and chirps (transient increases in EOD rate with waveform distortion). We calculated first off and first chirp latency as the time to first off/chirp minus the time of occurrence of the first attack. As EOD cessations are observed in both the contest and post-contest phase (Batista et al., 2012; Quintana et al., 2016), we calculated off rate as follows: (number of offs during contest + post-contest phase) divided by (contest duration + 600 s, the arbitrary recorded duration of the post-resolution phase). As chirps are late submissive electric displays observed after contest resolution (Batista et al., 2012; Quintana et al., 2016), we calculated chirp rate by dividing the number of post-contest chirps by 600 s.

### Statistics

All data were analyzed by non-parametric tests: Mann–Whitney *U*-test (independent variables using sets of data from different fish, or for comparing dominants vs. subordinates), and Wilcoxon paired test (paired variables using sets of data from the same fish). When comparing among three groups, we used Kruskal–Wallis test, and when significant changes were detected (*p* ≤ 0.05) we used *post-hoc* Dunn test to detect pairwise statistical differences. To test the effects of AVT and MC administration on contest outcome and the emission of transient electric signals, we used Chi-square tests 3 × 2 (χ2). Accordingly, data are expressed as median ± median absolute deviation (MAD) throughout. Statistics were calculated with Graphpad Prism 7, graphs were created with Origin 8.0 Pro, and figures were made with Inkscape 0.92.0.

## Results

All dyads of non-breeding *G. omarorum* tested (control, AVT, and MC-treated) displayed agonistic behavior immediately after the gate was removed, and dominance-subordination status was established within a few minutes (< 10 min in all cases, Table 1). All the agonistic encounters (control, AVT, and MC-treated) also showed similar temporal profiles and followed the typical 3 phases: (a) a short pre-contest of around 30 s; (b) the contest, characterized by highly aggressive displays by both contenders; and (c) the 10-min post-contest phase, in which dominants persisted in attacking, while subordinates attempted to flee and emitted submissive electric signals. Contest outcome was predictable by body weight asymmetry and no significant outcome reversion was observed by any of the pharmacological manipulations (control, AVT, and MC-treated, Chi square test, *p* > 0.05, Table 1).

**Figure 1:**
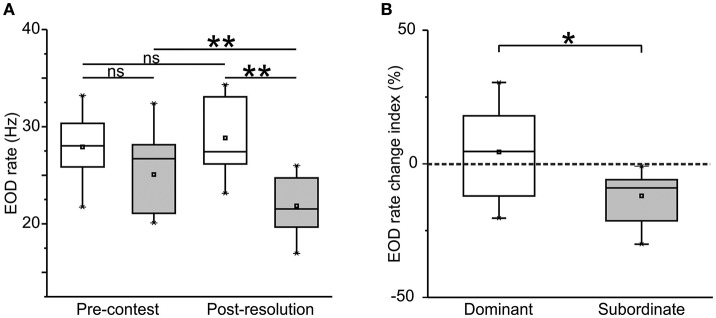
Electric submission. **(A)** EOD rate of dominants (white) and subordinates (gray) in pre-contest and post-resolution phases of agonistic encounters. Pre-contest dominants vs. pre-contest subordinates, Mann–Whitney *U*-test, *p* = 0.15. Post-resolution dominants vs. subordinates, Mann–Whitney *U*-test, *p* = 0.0002. Pre-contest vs. post-resolution dominants, Wilcoxon paired test, *p* = 0.58. Pre-contest vs. post-resolution subordinates, Wilcoxon paired test, *p* = 0.001, *n* = 11 dyads. **(B)** EOD rate change index. Values around 0 (dotted line in graph) indicate no change in the EOD rate. Index values show a significant decrease in the EOD rate of subordinates whereas the EOD rate of dominants did not change. Index dominants vs. index subordinates, Mann–Whitney *U*-test, *p* = 0.03, *n* = 11 dyads. Box chart symbols: Mean (square), median (line in the middle), 25–75% interquartile range (lower and upper borders), minimum, and maximum values (lower and upper error bars). ^*^p < 0.05; ^**^p < 0.01.

*Gymnotus omarorum* signals the dominance-subordination status by EOD rate rank. As shown in Figure 1A, subordinates decreased their EOD rate after contest resolution, and thus showed a negative EOD rate change index [Figure 1B, −9.04 (±6.54)]. Dominants, on the other hand, did not change their EOD rate during the contest (Figure 1A) and showed an EOD rate change index close to zero [Figure 1B, 4.62 (±13.44)]. Whereas, EOD rate was indistinguishable between the contenders during the pre-contest phase, the EOD rate of the subordinates was significantly lower than that of their respective dominant after the agonistic encounter. An electric submission was therefore observed, evidenced by a different EOD rate change index between contenders (Figure 1B, EOD rate change index of dominants vs. EOD rate change index of subordinates, *p* = 0.03, Mann–Whitney *U*-test).

### Vasotocinergic modulation of aggression

As previously reported (Zubizarreta et al., 2015; Quintana et al., 2016), contenders of *G. omarorum* engaged in highly aggressive fights. Both dominants and subordinates of control dyads exhibited aggressive displays during the contest phase, though dominants' attack rate was always higher than subordinates' [Dominants: 0.119 (±0.045), Subordinates: 0.04 (±0.02), Mann–Whitney *U*-test, *p* = 0.0001]. The asymmetry of the intensity of aggression displayed after contest resolution was even more outstanding; when subordinates decreased their attacks to zero, dominants persisted in attacking with the same intensity [dominants' contest attack rate: 0.119 (±0.045); dominants' post-resolution attack rate: 0.012 (±0.008), Wilcoxon paired test, *p* = 0.65].

The administration of AVT or MC to subordinates prior to the encounter did not induce any change in their aggression levels nor in their readiness to attack with respect to saline controls (Table 1). In contrast, in dominants, though AVT treatment did not induce changes in their attack rate among the experimental groups, the administration of the AVT antagonist (MC) induced a decrease in the total attack rate with respect to saline ontrols, indicating the role of endogenous AVT in dominants' aggression (Table 1).

### Vasotocinergic modulation of agonistic electric displays

#### Rank-related EOD rate

**Figure 2:**
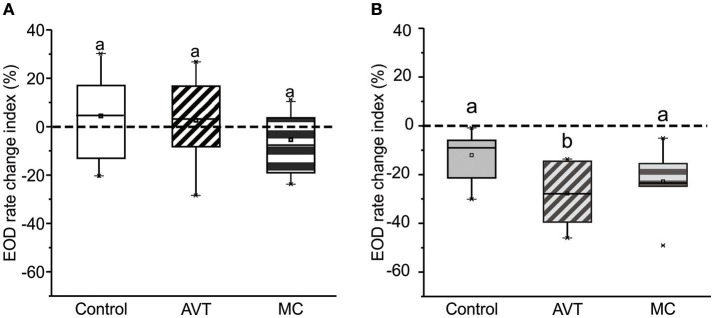
AVT effects on EOD rate change index. **(A)** Dominants. Neither AVT nor MC treatment affect the EOD rate change index in dominants. Kruskal–Wallis test, *p* = 0.46. ncontrol = 11, nAVT = 11, nMC = 6. **(B)** Subordinates. The EOD rate change index of subordinates after AVT treatment is more pronounced with respect to both saline subordinate controls and MC-treated subordinates. Kruskal–Wallis test, *p* = 0.04. *Post-hoc* Dunn test, Control vs. AVT, *p* = 0.04; Control vs. MC, *p* = 0.32; AVT vs. MC, *p* > 0.99. ncontrol = 11, nAVT = 9, nMC = 6. Dotted line indicates no change in EOD rate. Lowercase letters show statistically significance: same letter means non significant differences; different letters mean significant differences.

Neither AVT nor MC administration to dominants before the contest modified their EOD rate change index, which remained close to zero in all these experimental conditions [Figure 2A, Control: 4.62 (±13.44); AVT: 3.19 (±10.52); MC: −3.38 (±11.26), Kruskal–Wallis test, *p* = 0.46]. In contrast, the post-resolution EOD rate decrease observed in AVT-treated subordinates was ~3 times larger than the one observed in control subordinates (Figure 2B). While AVT administered to subordinates evoked an increase in their electric submission in comparison to saline controls, MC did not show a significant effect [Figure 2B, Control: −9.04 (±6.54); AVT: −27.66 (±11.79); MC: −21.18 (±4.64); Kruskal–Wallis test, *p* = 0.04. *Post-hoc* Dunn test, Control vs. AVT, *p* = 0.04; Control vs. MC, *p* = 0.32; AVT vs. MC, *p* > 0.99].

#### Transient social electric signals

**Figure 3:**
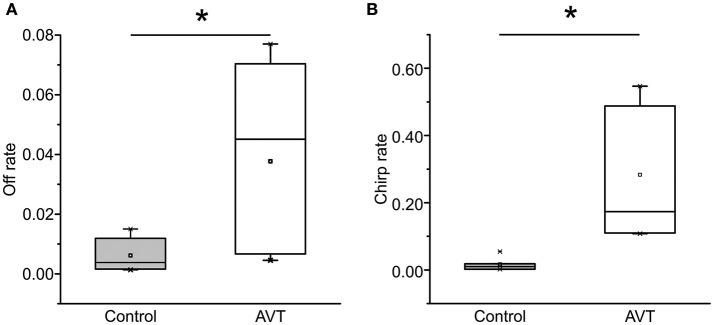
AVT effects on the rate of emission of transient electric submission signals. **(A)** Offs. AVT administered to subordinates increases off rate. Control vs. AVT, Mann–Whitney *U*-test, *p* = 0.03, ncontrol = 7, nAVT = 7. **(B)** Chirps. AVT administered to subordinates increases chirp rate. Control vs. AVT, Mann–Whitney *U*-test, *p* = 0.04. ncontrol = 7, nAVT = 6. ^*^p < 0.05.

The percentage of dyads in which subordinates produced either offs or chirps did not change with AVT nor MC treatment to subordinates, (offs: χ^2^ test 3 × 2, Control vs. AVT vs. MC, *p* = 0.72; chirps: χ^2^ test, 3 × 2, Control vs. AVT vs. MC, *p* = 0.56). Based on these results, we compared the rate of both offs and chirps between the dyads that actually produced these electric traits. As shown in Figure 3A, overall off rate was significantly increased after AVT administration [Control: 0.004 (±0.002), AVT: 0.045 (±0.032), Mann–Whitney *U*-test, *p* = 0.03, ncontrol = 7, nAVT = 7]. AVT administered to subordinates also produced a significant increase in post-resolution chirp rate [Figure 3B, Control: 0.01 (±0.008), AVT: 0.22 (±0.11), Mann–Whitney *U*-test, *p* = 0.001, ncontrol = 7, nAVT = 6]. The MC group did not present enough dyads that produced offs and chirps to carry out this statistical test.

## Discussion

It has been postulated that the control of social behavior in vertebrates relies on the activity of a conserved neural network (Newman, 1999; Goodson and Kabelik, 2009; O'Connell and Hofmann, 2011, 2012). A general prediction of the social behavior network hypothesis (Newman, 1999) is that a distinctive spatio-temporal pattern of activity of the network corresponds to each type of behavior, and that this network modifies its activity according to social contexts and physiological states within individuals. As a particular test of this prediction, we indirectly confirmed that the non-breeding territorial aggression displayed by dominants and subordinates of *G. omarorum* is controlled by two different activation patterns of the AVT system, revealing a status-dependent AVT modulation of aggression. Taking advantage of the asymmetry of locomotor and electric displays between contenders, our behavioral and pharmacological results suggest a finely tuned activation of the AVTergic system acting at different levels of the CNS in dominants and subordinates. In dominants, AVT most likely acts on the circuits modulating overt aggression; whereas in subordinates, AVT actions are directed to the electrogenic pathway modulating submissive electric displays.

AVT and AVP are recognized modulators of social behavior integrating external and internal cues (Insel and Young, 2000; Goodson and Bass, 2001; Godwin and Thompson, 2012). As one of its roles in social behavior, AVT is known to regulate aggression throughout vertebrates acting in a species-specific and context-dependent manner (Ferris et al., 1986; Goodson and Bass, 2001; Semsar et al., 2001; Goodson et al., 2009; Huffman et al., 2015; Terranova et al., 2016). However, only few studies across vertebrates demonstrate a distinctive AVT modulation between dominants and subordinates from pharmacological experiments. In the violet-eared waxbill, AVT supports male competition aggression in dominants but inhibits aggression in subordinates (Goodson et al., 2009); in the teleost bluehead wrasse, AVT inhibits aggression of territorial males but enhances aggression of non-territorial ones (Semsar et al., 2001). The non-breeding territorial aggression of *G. omarorum* provides the so far clearest example of non-overlapping status-dependent effects of AVT: while in dominants AVT promotes aggression without affecting their electric displays; in subordinates, AVT induces an increase in the emission of electric submissive displays without affecting their locomotor aggression levels.

### AVT enhances electric signaling of submission

Communication signals that convey fighting ability, submission, threat, and/or social rank by non-aggressive means enable contestants to avoid costly fights when the outcome is predictable (Preuschoft and van Schaik, 2000). The use of electrocommunication signals for this purpose has been demonstrated in several electric fish species within the agonistic context, beginning with pioneering behavioral experiments in the 1970's (Black-Cleworth, 1970; Westby G., 1975; Hagedorn and Heiligenberg, 1985; Zakon et al., 2002; Perrone et al., 2009; Batista et al., 2012).

As an integrator of social behavior, AVT modulates communication systems in many groups of vertebrates; e.g., in the vocal circuitry of the plainfin midshipman (*Porichthys notatus*), a teleost fish that uses social vocalizations in multiple behavioral contexts, (Goodson and Bass, 2000; Boyd, 2013); or in advertising calling behavior of the coqui frog (Ten Eyck, 2005). Using artificial stimuli presented to confined fish, several previous studies in weakly electric fish have demonstrated that AVT regulates the emission of chirps in *Apteronotus leptorhynchus* (Bastian et al., 2001), interruptions in *Eigenmania virescens* (Wong, 2000), and EOD basal rate in *G. omarorum* and *Brachyhypopomus gauderio* (Perrone et al., 2010, 2014). In agonistic context, endogenous AVT has been recently demonstrated as being responsible for the EOD rate rank dominance of *B. gauderio* (Perrone and Silva, 2016). Our present study in the agonistic behavior of *G. omarorum* is the first to demonstrate status-dependent actions of AVT on the electric signaling of contenders, as the same AVT treatment induced clear changes in subordinates but no changes in dominants. Though the involvement of AVT in the modulation of submissive electric signaling confirms the context-dependency of its effects, it was somehow expected, since in control encounters only subordinates modulate their electric discharges to signal submission (Batista et al., 2012; Quintana et al., 2016).

As reported previously in other electric fish species (Hopkins, 1974; Westby G. W. M., 1975; Hagedorn and Heiligenberg, 1985; Zakon et al., 1991; Triefenbach and Zakon, 2008; Fugère et al., 2010; Perrone and Silva, 2016), *G. omarorum* establishes an electric social rank after agonistic encounters. In contrast to the reproductive male aggression of *B. gauderio* (Perrone and Silva, 2016), in the territorial aggression of *G. omarorum*, the subordinate fish decreases its EOD rate after contest resolution to signal submission electrically (Figure 1). Rank-related EOD rate modulations in *G. omarorum* are controlled by the AVTergic system as AVT administration exacerbates electric submission; i.e., of the post-resolution decrease of EOD rate observed in subordinates (Figure 2B). It is important to mention that this is the first observation in weakly electric fish of inhibitory AVT actions on EOD rate. In *B. gauderio*, AVT mediates the EOD rate increase observed in dominants after contest resolution (Perrone and Silva, 2016). Accordingly, we have previously demonstrated in *B. gauderio* that the EOD basal rate and the additional nocturnal increase observed in dyads during breeding season are AVT-dependent (Perrone et al., 2010). In isolated diurnal *G. omarorum*, AVT administration also induces a transient and small increase in EOD rate that can be mimicked by AVT administration in brain slices containing the medullary pacemaker nucleus (Perrone et al., 2014).

In this study, we demonstrated a very clear example of context-dependent action of AVT; only in subordinates, AVT has an inhibitory action on EOD rate reinforcing the signaling of submission. AVT injection to the potential subordinate not only enhances electric submission (as discussed above) but also increases off and chirp rates (Figure 3). Unfortunately, we were unable to demonstrate the endogenous role of AVT on electric submission displays as MC did not reverse the effects of AVT on subordinates. However, as this lack of effect may be due to the MC dose we used, this study still allows us to speculate on a distinctive role of AVT on subordinates. Moreover, our results suggest separate actions of AVT modulation at different levels of the electrogenic system in the same species. That is, modulation of EOD basal rate (electric submission) suggests a direct or indirect AVT action on the medullary pacemaker nucleus, whereas modulation of chirps and interruptions more likely implies direct or indirect AVT actions on mid-brain pre-pacemaker structures (Kawasaki et al., 1988; Kawasaki and Heiligenberg, 1989; Heiligenberg et al., 1991; Keller et al., 1991). Based on these results, we can postulate, that the AVT system contributes to the adoption of a subordinate configuration of the electric communication system.

### AVT modulation supports overt aggression in dominants

Despite their context-dependent actions, AVT/AVP are considered as promoters of aggression across vertebrates (Ferris and Potegal, 1988; Stribley and Carter, 1999; Semsar et al., 2001; Larson et al., 2006; Santangelo and Bass, 2006; Goodson and Kabelik, 2009; Kabelik et al., 2009; Veenema et al., 2010). In the present study, we contribute evidence to this reported action of AVT as the pharmacological manipulations of the AVT system performed in dominants of *G. omarorum* alter their overt aggression (Table 1). The evidence that MC administration to dominants induces a decrease in their levels of aggression compared to control, implying that AVT is probably secreted in dominants during the agonistic contest, and reinforces the role of endogenous AVT in the control of aggression in dominants. Further, our data suggest that this endogenous tone of dominants' AVT has already induced a maximum plateau of aggression intensity that cannot be further enhanced by exogenous AVT administration. In the only previous report among teleosts in which the effects of exogenous AVT administration on aggression levels were evaluated in dominants and subordinates of the same species (Semsar et al., 2001), opposite status-dependent effects on aggression were confirmed. It is important to note that this is not the case for the agonistic behavior of *G. omarorum* as the pharmacological manipulations (either AVT or MC administration) induce changes in different systems, affecting the locomotor and aggressive displays in dominants (Table 1), but the electric signaling in subordinates (Figures 2, 3). It is also noteworthy, that in contrast to previous reports (Ferris, 1992; Huffman et al., 2015), no treatment caused contest outcome reversion.

Status-dependent activation of the AVT system between dominants and subordinates has been already postulated in teleosts. A common dual organization of two populations of preoptic AVT neurons (giganto-magnocellular vs. parvocellular) has been postulated, in which aggressive (often territorial) species have larger AVT-ir cells within the gigantocellular preoptic cell group than non-aggressive species (Greenwood et al., 2008; Dewan et al., 2011; Godwin and Thompson, 2012). Within the same species, several studies have found morphometric and functional differences among AVT cell-groups related to the dominant-subordinate status (Larson et al., 2006; Greenwood et al., 2008; Iwata et al., 2010; Dewan and Tricas, 2011; Ramallo et al., 2012; Almeida and Oliveira, 2015; Loveland and Fernald, 2017). A recent report has proven that in zebrafish, dominants. and subordinates have different AVT levels in several areas of the social brain (Teles et al., 2016). Accordingly, new findings of our group show a differential activation of AVT neurons at the POA between dominants and subordinates during the agonistic encounter of *G. omarorum* (Pouso, 2017).

## Final remarks

Dyadic agonistic encounters essentially result in asymmetric behaviors, in which the displays of dominants and subordinates are theoretically controlled by distinctive patterns of activation of the social brain network nuclei. However, at least in teleosts, pharmacological approaches have failed to give a comprehensive view of status-dependent AVT modulation. The rich repertoire of locomotor and electric traits of the non-breeding territorial aggression of *G. omarorum* allowed us to put forth clear evidence of asymmetric strategies of AVT modulation between dominants and subordinates. The AVTergic system does not appear to contribute to determining the contest outcome in *G. omarorum*. Rather, it seems likely that the AVTergic system adopts two distinctive configurations that participate in the consolidation of either the dominant or the subordinate status. Taken together, our data indicate that AVT modulation of agonistic behavior in *G. omarorum* acts in a non-overlapping status-dependent manner. In contrast to other species, in which opposite actions of AVT among contenders were reported on the same trait (e.g., aggression levels), in *G. omarorum*, AVT affects independent displays in dominants and subordinates. In dominants, AVT regulates the intensity of aggression but does not affect any electric display; while in subordinates, AVT enhances the electric signaling of submission without affecting any locomotor display. This study contributes the clearest example among teleosts of status-dependent neuropeptidergic modulation, by demonstrating different actions of AVT in the agonistic behavior of dominants and subordinates.

## Author contributions

RP: Designed and performed the experiments, analyzed the data, participated in the discussion of results, wrote the paper, contributed reagents and equipment. All the experiments and results were part of RP's Ph.D. thesis; AS: Designed and supervised the experiments, participated in the discussion of results, wrote the paper, contributed reagents, and equipment.

### Conflict of interest statement

The authors declare that the research was conducted in the absence of any commercial or financial relationships that could be construed as a potential conflict of interest.
